# Non-Destructive Detection of Tea Leaf Chlorophyll Content Using Hyperspectral Reflectance and Machine Learning Algorithms

**DOI:** 10.3390/plants9030368

**Published:** 2020-03-17

**Authors:** Rei Sonobe, Yuhei Hirono, Ayako Oi

**Affiliations:** 1 Faculty of Agriculture, Shizuoka University, Shizuoka 422-8529, Japan; 2 Institute of Fruit Tree and Tea Science, National Agriculture and Food Research Organization, Shimada 428-8501, Japan

**Keywords:** deep belief nets, extreme learning machine, first derivative spectra, random forest, shade-grown tea, support vector machine

## Abstract

Tea trees are kept in shaded locations to increase their chlorophyll content, which influences green tea quality. Therefore, monitoring change in chlorophyll content under low light conditions is important for managing tea trees and producing high-quality green tea. Hyperspectral remote sensing is one of the most frequently used methods for estimating chlorophyll content. Numerous studies based on data collected under relatively low-stress conditions and many hyperspectral indices and radiative transfer models show that shade-grown tea performs poorly. The performance of four machine learning algorithms—random forest, support vector machine, deep belief nets, and kernel-based extreme learning machine (KELM)—in evaluating data collected from tea leaves cultivated under different shade treatments was tested. KELM performed best with a root-mean-square error of 8.94 ± 3.05 μg cm^−2^ and performance to deviation values from 1.70 to 8.04 for the test data. These results suggest that a combination of hyperspectral reflectance and KELM has the potential to trace changes in the chlorophyll content of shaded tea leaves.

## 1. Introduction

Green tea, which is produced from unfermented tea leaves, is widely consumed in East Asia and has recently gained popularity in the West [1]. Consumption of matcha (powdered green tea) or matcha-flavoured sweets has increased to the extent that Japan’s green tea exports reached approximately USD 14.1 billion in 2018 [2]. The chlorophyll content is strongly correlated with the colour of dry tea leaves [3], with a high chlorophyll content improving the tea leaf appearance. Shade treatment reduces plant transpiration compared with plants in full photosynthetic photon flux density, but it also reduces photosynthesis sensitivity [4] and tends to increase leaf nutrient concentrations and leaf chlorophyll content [5]. Based on this phenomenon, in Shizuoka prefecture, Japan, shade nets (70%–95% shading) are used to control light transmission for 14 to 30 days before picking, thereby increasing the leaf chlorophyll content [6,7]. While, traditionally, only first-grade tea (from the first picking) for producing tencha (tea used for preparing matcha) has been shade-treated, the application of shade treatment for the second picking of tea to increase matcha production has recently been implemented in Shizuoka, Japan [6]. However, the excessive environmental stresses caused by low light conditions sometimes lead to early mortality of tea trees [7]. Thus, techniques are required for better management of tea trees, for quantifying chlorophyll content, and for detecting environmental stresses [8].

Ultraviolet and visible light (UV–vis) spectroscopy and high-performance liquid chromatography (HPLC) techniques have previously been used to quantify chlorophyll content. These are destructive methods and may enforce a limited sample size because they are expensive and labour-intensive. It is also not always possible to apply these methods in situ. Alternately, some portable chlorophyll content meters, such as CL-01, SPAD-502, Dualex, and CCM-200, have been used to estimate chlorophyll content [9]. However, light intensity also influences leaf thickness [10], and then it often makes the output of the meter obscure [11]. In contrast, hyperspectral reflectance measurements may be used for detecting various responses from crops [12] or evaluating vegetation properties [13,14], and the use of reflectance for estimating chlorophyll content is being seriously considered. Some pigments, such as chlorophylls and carotenoids, absorb energy strongly in the ultraviolet, blue, and red regions, and then the reflectance and transmittance are weak [15]. The reflectance from vegetation is characterised by the low reflectance over the blue (400–500 nm) and red (650–690 nm) spectral regions due to strong absorption by chlorophylls plus carotenoids in the blue wavelengths and chlorophyll *a* in the red wavelengths, and the steep rise in reflectance between 680 and 750 nm is caused by the chlorophylls increasing to absorb at wavelengths beyond 700 nm [16]. Based on these features, some empirical approaches, such as vegetation indices, which are generally based on a few narrow or broad spectral bands, are convenient for use to estimate chlorophyll content. Most vegetation indices for chlorophyll content use wavelengths ranging from 400 to 860 nanometres [17,18,19] or the red edge (680 to 750 nm) [20,21]. However, some datasets, such as LOPEX, CALMIT, ANGERS, and HAWAII, that have been used for the development of vegetation indices, were collected under relatively low light-stress conditions. Their coefficients of linear regression for estimating chlorophyll *a* content from carotenoid contents range from 2.99 (for the LOPEX dataset) to 3.45 (for the HAWAII dataset) [22]. Further, some proposed techniques are not appropriate for evaluating the chlorophyll content of shade-grown tea, since shade treatment imposes environmental stress on vegetation and alters the relative content as well as the allocation of chlorophyll and carotenoids. Furthermore, a shift of the red edge toward longer wavelengths (red shift) and a shift toward shorter wavelengths in the 700–780 nm region (blue shift) are caused by environmental stresses [23], and more shading makes the reflectance lower at the red edge inflection point (REIP) in tea cultivation [6].

Using machine learning algorithms is effective for expressing complex relationships [24]. Deep learning algorithms, in particular, have been used for classification purposes, and the convolutional neural networks-based approach is effective for detecting plant diseases [25]. This methodology also has potential in the analysis of hyperspectral reflectance data. Deep belief nets (DBN) is a probabilistic generative model composed of multiple layers of stochastic latent variables [26] that has performed well when using hyperspectral remote sensing [27]. Random forest (RF), another machine learning algorithm, is a regression technique that combines numerous decision trees to classify or predict the value of a variable. It has been used for estimating vegetation properties [28,29,30] as well as for classification and regression [31]. Support vector machine (SVM) is an approach used with a Gaussian kernel function [32] that is expedient for identifying the relationship between reflectance and leaf water status [33]. In addition to these three techniques, the kernel-based extreme learning machine (KELM), which uses only two hyperparameters, performs well in fault diagnosis, recognition, classification, and signal processing [34].

The use of machine learning algorithms requires tuning of their hyperparameters. The determination of hyperparameters corresponds to a convex optimization problem [35]; traditionally, grid search strategies have been applied as a solution [36]. However, such strategies are poor choices for configuring algorithms for new data sets, in which case a random search is thought to be a superior approach [37]. Previous studies have demonstrated that Bayesian optimisation (a framework used to optimise hyperparameters of noisy, expansive black-box functions that constitutes a structured approach to modelling uncertainty) performs even better [38]. A Gaussian process (GP) is commonly used for Bayesian optimisation [39]. Recently, approaches using extreme learning machines (ELMs) or neural networks have been given consideration for generating regression models [40,41]. Although there are several machine learning algorithms that are currently used in hyperspectral remote sensing, to date, there has been no consensus regarding the best algorithm, and a comparison of different algorithms is thus beneficial.

The first derivative of reflectance has been used to detect specific points such as the green peak and the red edge inflection point (REIP) [42]. Various hyperspectral indices based on the first derivative spectra have been recommended for evaluating vegetation properties [43,44,45,46,47], since the first derivative spectra may enhance weak spectral features which are effective for evaluating target parameters [48]. Therefore, the first derivative spectra were also evaluated.

The main objectives of this study are (1) to evaluate the potential of hyperspectral data, including reflectance and first derivative spectra, for estimating the chlorophyll content of tea leaves cultivated in full sunlight (0% shading) and low-light (shaded) conditions and (2) to identify which algorithms are the most suitable for constructing regression models from these data.

## 2. Results

### 2.1. Chlorophyll Content, Spectral Reflectance, and Their Correlations

**Figure 1:**
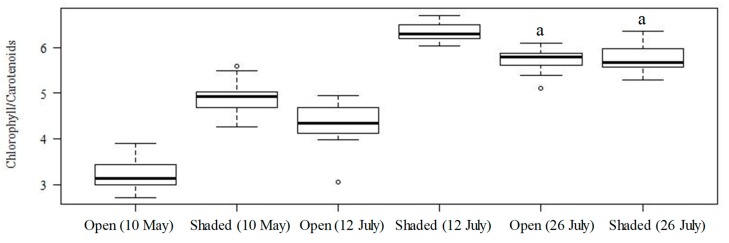
Boxplots of the ratio of total chlorophyll to carotenoids. Values with the same letter (i.e., a) are not significantly different (*p* < 0.05).

Chlorophyll content was measured based on the absorbance of the supernatant dimethyl-formamide extract (Table 1). The mean values of chlorophyll content per leaf area (μg cm^−2^) were 53.77 and 74.92 on 10 May, 47.07 and 46.25 on 12 July, and 111.93 and 95.32 on 26 July for 0% and 85% shading, respectively (Table 1). The difference in chlorophyll content was only significant between the treatments on 10 May, although a Tukey–Kramer test showed that shading was not effective for increasing the chlorophyll content of second picking leaves. In fact, this treatment prevented increases in chlorophyll content and merely resulted in an increased difference (albeit not significantly so). Figure 1 shows the chlorophyll/carotenoid ratios calculated for the different picking dates and shade treatments. While ratios ranged from 2.71 to 3.91 for the unshaded samples picked on 10 May, they were greater than 4.00 for all other samples (barring some irregular values).

**Figure 2:**
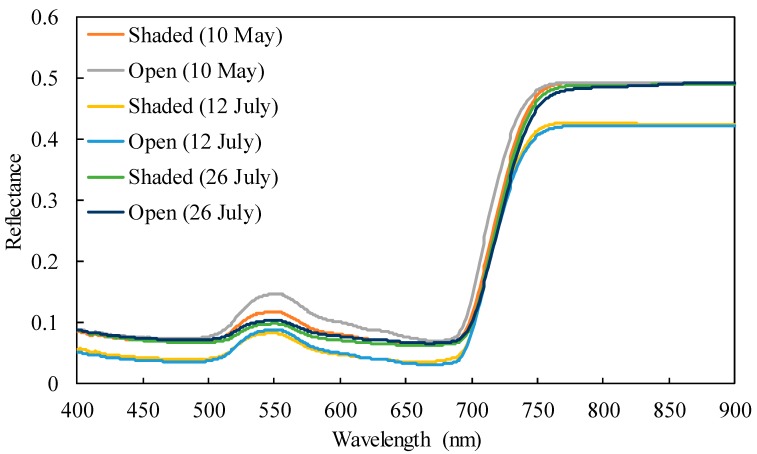
Mean reflectance spectra.

Figure 2 shows the mean reflectance for each date and treatment. A comparison of leaves from the first picking (10 May) showed that shading pushed the reflectance near to the green peak and lower at the red edge inflection point (REIP). Further, the reflectance of the 0% and 85% shaded samples separated completely at certain wavelengths (e.g., 725 nm). However, these tendencies were not observed during subsequent pickings. Reflectance values at some wavelengths were required to separate the samples of the two treatments. Thus, reflectance values at 400, 414, 427, 437, 515, 692, and 780 nm and at 539, 698, and 752 nm were differentiated using a stepwise linear discriminant analysis for measurements obtained on 12 and 26 July, respectively.

**Figure 3:**
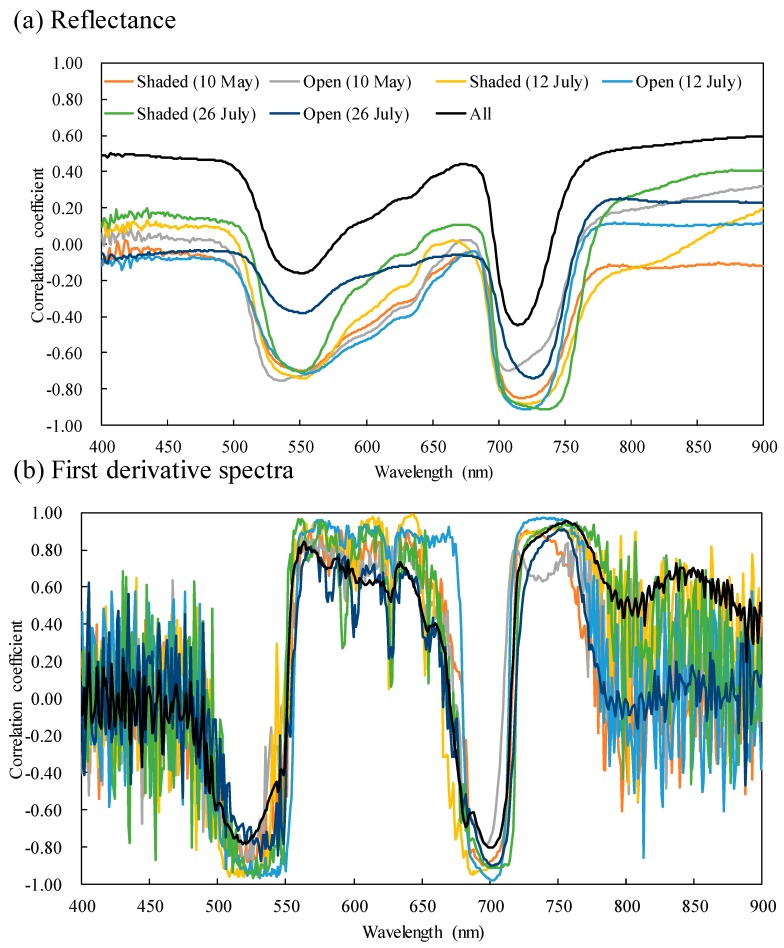
Correlations (**a**) between chlorophyll content and reflectance and (**b**) between chlorophyll content and first derivative spectra.

The correlation coefficients between the reflectance and chlorophyll content (Figure 3) revealed two troughs: one near the green peak (R = −0.70 at 552 nm for shaded leaves on 10 May; R = −0.75 at 535 nm for non-shaded leaves on 10 May; R = −0.74 at 553 nm for shaded leaves on 12 July; R = −0.71 at 555 nm for non-shaded leaves on 12 July; R = −0.70 at 550 nm for shaded leaves on 26 July; R = −0.38 at 551 nm for non-shaded leaves on 26 July) and the other near the REIP (R = −0.85 at 718 nm for shaded leaves on 10 May; R = −0.70 at 707 nm for non-shaded leaves on 10 May; R = −0.88 at 720 nm for shaded leaves on 12 July; R = −0.91 at 720 nm for non-shaded leaves on 12 July; R = −0.91 at 735 nm for shaded leaves on 26 July; R = −0.74 at 726 nm for non-shaded leaves on 26 July). With the exception of non-shaded leaves collected on 10 May, the reflectance near the REIP showed higher absolute values of correlation coefficients than those near the green peak. Furthermore, no clear correlation existed between reflectance near the green peak and chlorophyll content when all measurements were combined.

The first derivative spectra confirmed a positive correlation between the REIP and wavelengths that are slightly longer than at the green peak (between 550 and 650 nm) and a negative correlation between wavelengths that are slightly shorter than at the green peak (between 500 and 550 nm) and the start of the red edge domain. The highest positive correlations were confirmed at 576 (R = 0.92), 633 (R = 0.93), 644 (R = 0.99), 735 (R = 0.97), 562 (R = 0.96), and 755 nm (R = 0.91) and the highest negative correlations were confirmed at 533 (R = −0.91), 525 (R = −0.92), 515 (R = −0.96), 702 (R = −0.98), 526 (R = −0.97) and 702 nm (R = −0.90) for shaded leaves on 10 May, non-shaded leaves on 10 May, shaded leaves on 12 July, non-shaded leaves on 12 July, shaded leaves on 26 July, and non-shaded leaves on 26 July, respectively.

### 2.2. Performance of Machine Learning Approaches Using Original Reflectance and First Derivative Spectra

**Figure 4:**
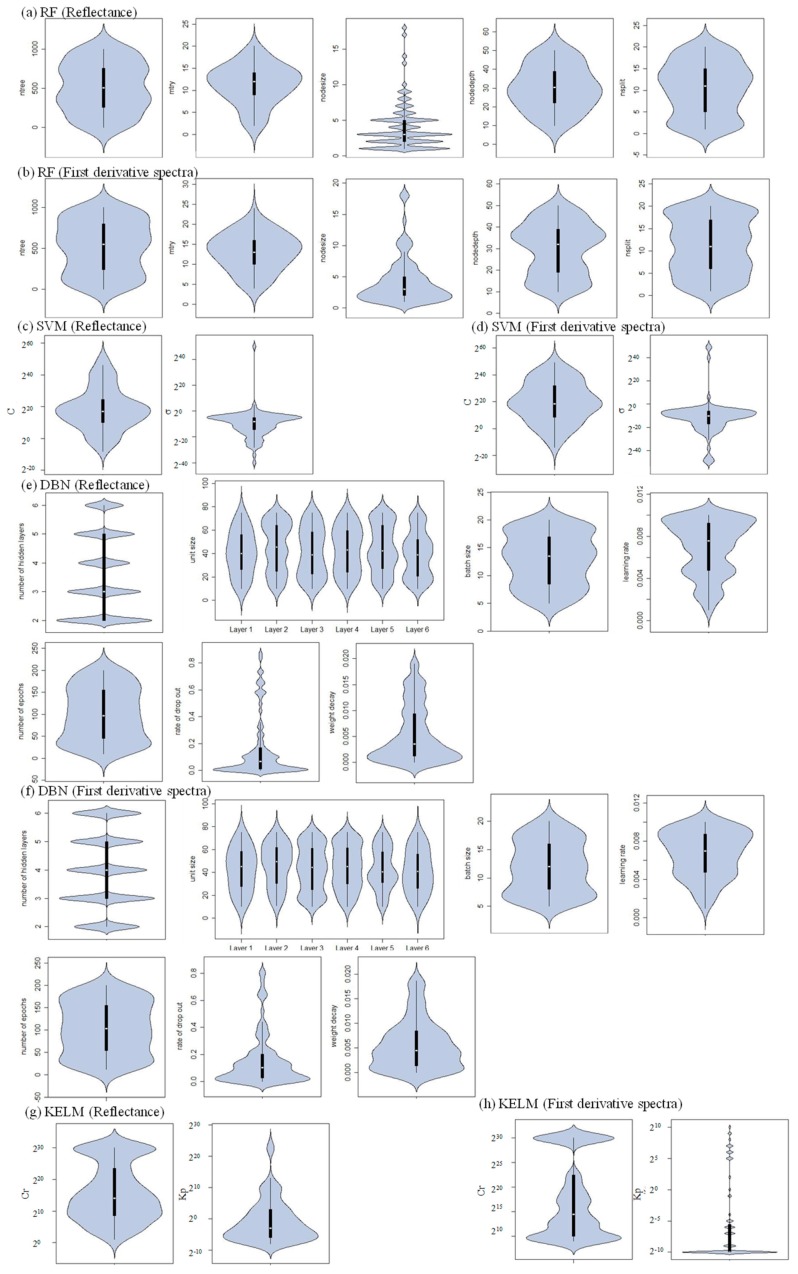
Distributions of selected hyperparameters based on Bayesian optimisation.

The optimal values of hyperparameters are shown in Figure 4. When the original reflectance was used, the mean values for combinations of hyperparameters were (ntree, mtry, nodesize, nodedepth, nsplit) = (513, 11, 4, 30, 10) for RF, (C, ϭ) = (2^18^, 2^−10^) for SVM, (number of hidden layers, unit size of first layer, unit size of second layer, unit size of third layer, unit size of the fourth layer, unit size of the fifth layer, unit size of the sixth layer, batch size, learning rate, number of epochs, rate of drop out, weight decay) = (4, 41, 45, 41, 43, 44, 39, 13, 0.006919, 100, 0.157386, 0.005528) for DBN, and (Cr, Kp) = (2^15^, 2^1^) for KELM, while (ntree, mtry, nodesize, nodedepth, nsplit) = (519, 13, 4, 30, 11) for RF, (C, ϭ) = (2^20^, 2^−12^) for SVM, (number of hidden layers, unit size of first layer, unit size of second layer, unit size of third layer, unit size of fourth layer, unit size of fifth layer, unit size of sixth layer, batch size, learning rate, number of epochs, rate of drop out, weight decay) = (4, 43, 46, 46, 44, 43, 41, 11, 0.006685, 104, 0.157487, 0.005637) for DBN, and (Cr, Kp) = (2^17^, 2^−6^) for KELM.

**Figure 5:**
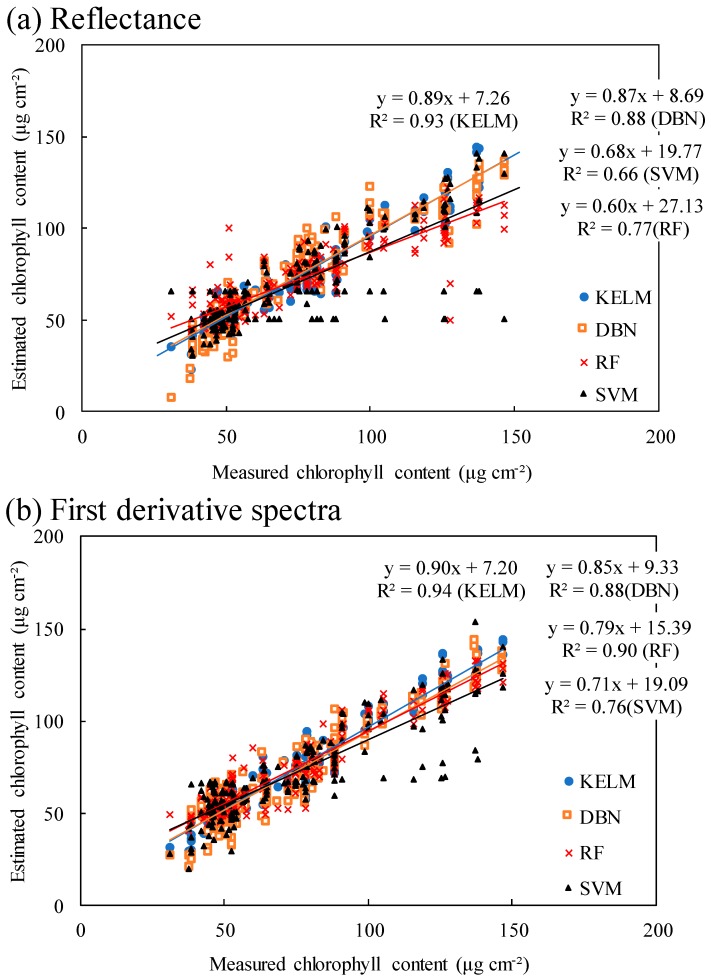
Relationships between estimated and measured chlorophyll contents.

**Figure 6:**
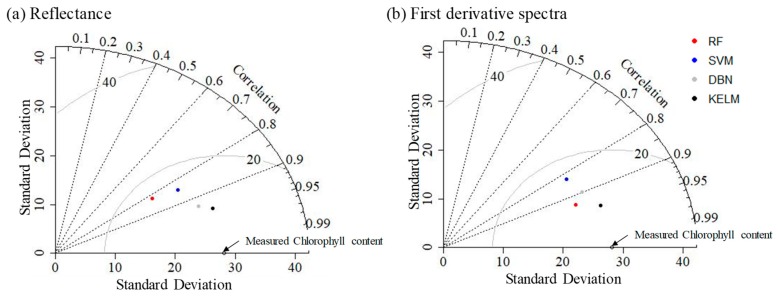
Taylor diagrams showing performances of regression models based on machine learning algorithms and (**a**) reflectance or (**b**) first derivative spectra. The grey counter indicates the root-mean-square error (RMSE) values. RF: random forest, SVM: support vector machine, DBN: deep belief nets, KELM: kernel-based extreme learning machine.

**Figure 7:**
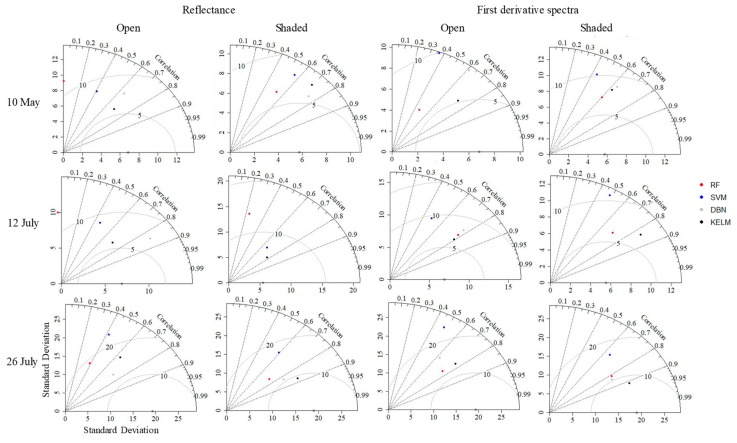
Taylor diagrams showing the performance of regression models for each treatment (open or shaded tea trees). RF: random forest, SVM: support vector machine, DBN: deep belief nets, KELM: kernel-based extreme learning machine.

Figure 5 shows the relationships between estimated and measured chlorophyll contents, the statistics of RPD (ratio of performance to deviation) and RMSE values calculated using regression models based on machine learning algorithms are shown in Table 2, and the performances of regression models based on machine learning algorithms are shown in Figure 6 and Figure 7. For RF and KELM, derivative spectra values were effective for estimating chlorophyll content rather than reflectance values, while the other two algorithms had smaller RMSEs when reflectance was used. For all algorithms, the mean values of RPD were greater than 1.4 (Category B) when original reflectance values were applied, which indicates that all resulting regression models were acceptable for estimating chlorophyll content. Mean RPD values were greater than 2.0 (Category A) when derivative spectra values were used. For all 100 repeats, DBN and KELM displayed RPD values of greater than 1.4 when reflectance data were used. When derivative spectra values were used, KELM was still acceptable for all 100 repeats; however, DBN occasionally produced RPD values below 1.4 (four instances).

### 2.3. Sensitivity Analysis

**Figure 8:**
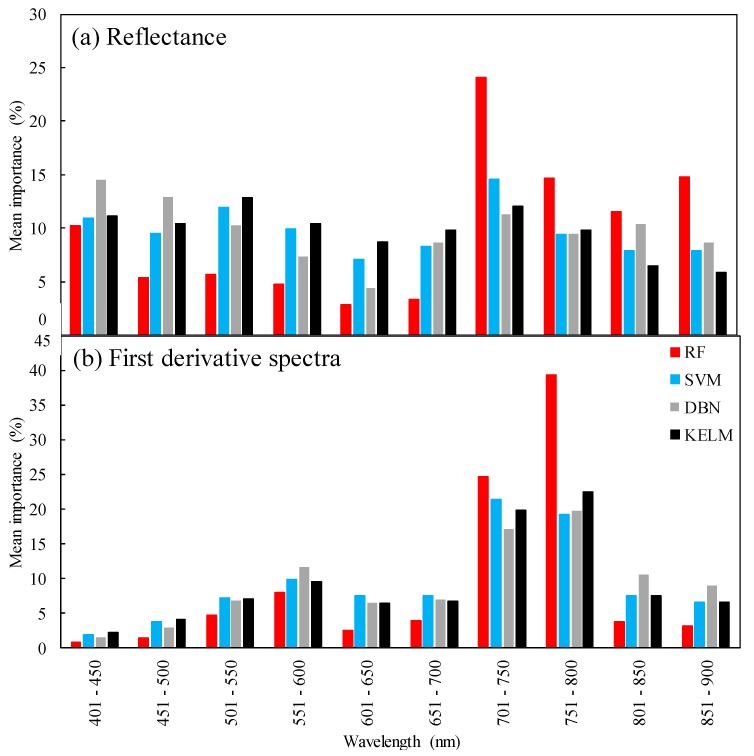
Data-based sensitivity analyses (DSA) results for random forest (RF), support vector machine (SVM), deep belief nets (DBN), and kernel-based extreme learning machine (KELM) from (**a**) reflectance and (**b**) first derivative spectra. Values were averaged over 100 replicates.

The DSAs (data-based sensitivity analyses) showed similar patterns of importance among the different algorithms (Figure 8). The highest importance values were confirmed at 701–750 nm for SVM and at 751–800 nm for RF, DBN, and KELM when first derivative spectra were used. The reflectance at 701–750 nm still had the greatest influence on chlorophyll content estimation; however, its importance was mostly unclear, except when using the RF technique and near the green peak for SVM and KELM.

## 3. Discussion

### 3.1. Characteristics of Leaf Samples Based on Photosynthetic Pigment Contents

In higher plants like tea trees, chlorophyll pigment consists of chlorophyll *a* and chlorophyll *b*. Concentrations relate closely to primary production because these pigments absorb sunlight and use their energy to synthesize carbohydrates using CO_2_ and H_2_O [49]. While carotenoids are also involved in photoprotection and light collection during photosynthesis [50], they also help to protect unsaturated fatty acids, phospholipids, and galactolipids from oxidative damage [51]. Some previous studies showed that the chlorophyll *a*/*b* ratio increases linearly, and the ratio is positively correlated with the amount of the core complex of photosystem II [52,53]. On the other hand, carotenoids are also involved in photoprotection and light collection in photosynthesis [54], and they also help to protect unsaturated fatty acids, phospholipids, and galactolipids from damage [51]. Based on these features of photosynthetic pigments, the total chlorophyll/carotenoid ratio or chlorophyll *a*/carotenoid ratio have been used as good indicators for evaluating environmental stress in plants [8,22,55]. Generally, shaded leaves contain more photosynthetic pigments than leaves in sunlight, because such leaves increase their chlorophyll *a* content to allow themselves to harvest more light and nitrogen [56]. Thus, light stress increases the chlorophyll/carotenoid ratio. In this study, an experiment was conducted in a greenhouse, an environment that may stress tea trees over and above shade treatment.

### 3.2. Performance of Different Machine Learning Algorithms

Previous studies have evaluated the performance of random forest (RF) regression and reported that it possessed better results than stepwise regression and support vector machine (SVM), linear regression, and radiative transfer modelling [57] for estimating vegetation properties [58]. However, more suitable algorithms were found and kernel-based extreme learning machine (KELM) generally performed the best (49 and 57 out of 100 repetitions for reflectance and first derivative spectra, respectively) for estimating the chlorophyll content when assessed using the ratio of performance to deviation (RPD) values. Although SVM’s robustness has been reported in some studies [59,60,61], and it performed best in 20 and 37 of the repeats, it also showed the worst performance in 28 and 33 repetitions for the reflectance and first derivative spectra, respectively. These results strongly suggest that SVM is not a stable method. KELM and SVM are both kernel-based algorithms, and poor selection of kernel function parameters may negatively affect their accuracies [62]. Indeed, the variance of the kernel function parameters of KELM was apparently smaller than that of SVM. The selected the kernel bandwidth (σ) values (of the SVM-based approach) ranged from 2^−40^ to 2^50^ and from 2^−50^ to 2^49^, while the kernel parameter (Kp) ranged from 2^−8^ to 2^24^ and from 2^−10^ to 2^10^ for reflectance and first derivative spectra, respectively. Furthermore, ELM has fewer optimisation constraints [63], which has been shown to be an advantage in regression applications [64]. Although deep belief nets (DBN) has also been reported to have great performance [65] and it performed best in repetitions 30 and 6, its poor performance was also recognised. DBN had the maximum number of hyperparameters of the four algorithms examined in this study since a total of twelve hyperparameters (unit sizes of six layers, batch size, learning rate, number of epochs, rate of drop out and weight decay) had to be optimised. The relatively small training data set may have prevented the method from producing sufficient results for tuning its measured chlorophyll content for both reflectance and first derivative spectra. Further, the order of KELM, DBN, and SVM was the same for both the reflectance and first derivative spectra, although the similarity with the measured chlorophyll content decreased. The use of reflectance was more effective for this purpose due to its ability to recognise patterns, and the first derivative spectra were noisier than the original reflectance data (Figure 3), which might have prevented the machine learning algorithms from producing robust results. However, the models based on RF changed dramatically, and there was a clear advantage for using first derivative spectra. Generally, importance is focused on a small number of variables in RF-based models [6]. Its performance may be improved if the variables highly correlated with chlorophyll content are selected and if the effects from noisy variables (e.g., first derivative values from 400 to 500 nm and longer than 800 nm) are negligible.

### 3.3. Differences in Estimation Accuracy among Treatments

High correlation coefficients were confirmed between the measured values and estimated values from KELM or DBN, and irregular values were not confirmed for any data or treatment (Figure 4). On the contrary, RF-based methods produced less stressed samples (i.e., non-shaded samples on 10 May and 12 July). In this study, most of the samples were affected by shading treatments or the greenhouse, and this result implies that RF is unsuitable for imbalanced data caused by the small sample size of lower stressed measurements. SVM also had a similar feature, and its estimated values were almost constant in some combinations of training data, even though a stratified random sampling approach was applied (Figure 4). Generally, models based on KELM performed best for both reflectance and first derivative spectra for each treatment (open or shaded tea trees; Figure 6). However, those based on DBN performed best for the shaded samples collected on 10 May and for the unshaded samples collected on 26 July when reflectance was used. DBN tended to estimate the chlorophyll content with greater error since the sample sizes and standard deviations calculated from the chlorophyll content were small. If either of these issues is removed, DBN may, however, constitute the better option. It performed relatively well for the shaded samples collected on 26 July, which was the smallest sample collected.

Derivative spectra have been applied for quantifying the leaf chlorophyll content, and its great potential has been shown [21,44,45,66,67]. However, the first derivative spectra were only advantageous for the RF-based method since the accuracies of the other algorithms were reduced, or the differences in accuracy were small. Furthermore, although RF performed better for the shaded samples when reflectance data were used, this tendency was obscure for the first derivative spectra for all observations. RF was effective in certain bands because the importance concentrated on some specific bands (Figure 8). However, it is usually difficult to calculate first derivative spectra from the hyperspectral data obtained from satellite- or air-borne remote sensing, whose bandwidths are wider than those from FielSpec 4, limiting the applicability of this algorithm.

## 4. Materials and Methods

### 4.1. Measurements and Datasets

**Figure 9:**
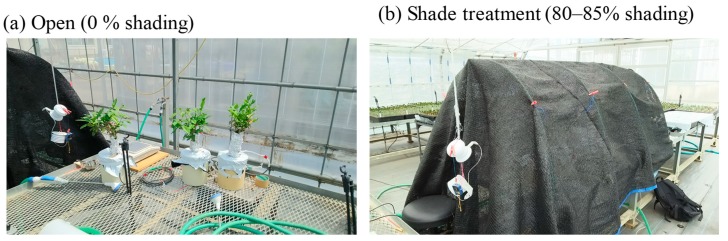
Shade treatments used during this study.

Our experiments were performed on six tea trees in a greenhouse at the Institute of Fruit Tree and Tea Science, National Agriculture and Food Research Organization, Shimada, Japan. Three trees were placed under a Dio Chemicals shading net #1800 (80%–85% shading, Dio Chemicals Ltd., Japan), while the remaining three trees were cultivated under 0% shading (unshaded; Figure 9), for the periods from 19 April to 10 May and 14 June to 12 July for the first and second flushes of leaves, respectively. After the first two pickings (sampled on 10 May and 12 July), all shade nets were removed, and the last samples were collected on 26 July. The numbers of samples are shown in Table 1.

New shoots were sampled from each tree on each sampling day, and reflectance and chlorophyll contents were measured from them. The numbers of samples collected differed between the two treatments since one tree and several leaves died before sampling.

An ASD FieldSpec4 unit (Analytical Spectral Devices, USA) was used to obtain reflectance data from leaf clippings. This device has three detectors: visible and near-infrared (VNIR), short wave infra-red (SWIR) 1, and SWIR 2. The splice correction function of ViewSpec Pro Software (Analytical Spectral Devices) was applied to correct differences in the spectral drifts (at 1000 and 1800 nm) caused by inherent variation in detector sensitivities. The first derivative spectra were also calculated from these reflectance spectra. After measuring reflectance, leaf discs (8 mm in diameter) were prepared and soaked in dimethyl–formamide to measure pigment concentrations using dual-beam scanning ultraviolet-visible spectrophotometers (UV-1280, Shimadzu, Japan). To quantify chlorophyll *a*, *b* and carotenoid contents (in μg ml^−1^) from the dimethyl–formamide extracts, the following calculations [68] were applied, and the results are expressed in μg cm^−2^:

(1) $$\mathrm{Car}=\left(1000A_{480}-1.12\mathrm{Chla}-34.07\mathrm{Chlb}\right)/245$$

(2) $$\mathrm{Chla}=12A_{663.8}-3.11A_{646.8}$$

(3) $$\mathrm{Chlb}=20.78A_{646.8}-4.88A_{663.8}$$

where A is the absorbance, and the subscripts represent the wavelength (nm).

For modelling, all measurements were divided into three groups (a training dataset (50%), a validation dataset (25%) and a test data dataset (25%)) using a stratified sampling approach [69] that was repeated a hundred times to obtain robust results.

### 4.2. Regression Model

When applying machine learning algorithms, it is necessary to select wavelengths that are effective for removing non-informative variables to obtain better and simpler prediction models [70]. The genetic algorithm (GA)-based approach is an adaptive heuristic search algorithm based on the concept of natural selection and survival of the fittest among individuals over consecutive generations. It was used to estimate the chlorophyll content because of its high performance in both regression and classification [70,71,72,73]. In this approach, each of five generations was composed of a population of character strings (i.e., combinations of narrow wavebands) analogous to a chromosome, from which the best waveband combination was finally selected after a process of evolution using R version 3.5.3 [74]. The regression models were then created using the selected bands and different supervised learning methods: RF, SVM, DBN, and KELM. For optimising the hyperparameters of these machine learning algorithms, Bayesian optimisation was applied with the Gaussian process [37,39] using R version 3.5.3 [74] and the “rBayesianoptimization” package [75].

#### 4.2.1. Random Forest (RF)

Random forest regression builds multiple decision trees called classification and regression trees (CART) based on randomly bootstrapped samples of the training data [76] via generalization of the binomial variance (using a Gini index) and with nodes that are split using the best split variable from a group of randomly selected variables [77]. Since previous research has demonstrated the effectiveness of RF [78,79], it was used as a benchmark in this study. The number of trees (ntree) and the number of variables used to split the nodes (mtry) are normally defined by the user. RF differs from CART in growing non-deterministically to decorrelate the trees and reduce variance using a two-stage randomisation procedure related to a bootstrap sample and random variable selection. Prior to the construction of each decision tree, several samples were extracted at random and replaced from the original training dataset; these samples were used for tree building. When ntree is increased, the generalisation error always converges; thus, over-training is not a problem. As a result, a tree of RF is grown as deeply as possible under the constraint that each terminal node must contain at least one case with node size ≥1. Furthermore, it is generally assumed that randomising the splitting rule can improve the performance of the ensembles [80]. Therefore, three additional hyperparameters were considered: the minimum number of unique cases in a terminal node (nodesize), the maximum depth to which a tree should be grown (nodedepth), and the number of random splittings (nsplit). RF regression was implemented using R version 3.5.3 [74] and the “randomForestSRC” package [81].

#### 4.2.2. Support Vector Machine (SVM)

Regression models based on SVM are effective for resolving the problems of high dimension and local minima [82], and SVM was used with the Gaussian radial basis function (RBF) kernel [83] using R version 3.5.3 [74] and the “e1071” package [84]. In this method, two hyperparameters, the regularisation parameter C and the kernel bandwidth σ, are tuned to generate regression models. For C, high values could result in over-fitting due to high penalties for inseparable points, while low values might lead to under-fitting. The σ value defines the reach of a single training example.

#### 4.2.3. Deep Belief Nets (DBN)

Deep belief net modelling consists of multi-layer, unsupervised, restricted Boltzmann machines (RBMs), which are two-layer neural networks [85]. Dropout is used during the training phase since this is known to facilitate good predictions. Eleven hyperparameters (unit sizes of six layers, batch size, learning rate, number of epochs, rate of drop out and weight decay) were optimised in this study. DBN regression was implemented using R version 3.5.3 [74] and the “darch” package [86].

#### 4.2.4. Kernel-Based Extreme Learning Machine (KELM)

The extreme learning machine (ELM) is based on a single hidden layer feedforward neural network. Its input weights and hidden layer biases are randomly assigned [87]. ELM has been successfully used in prediction, fault diagnosis, recognition, classification, and signal processing [34]. The kernel trick was applied to ELM instead of attempting to fit a non-linear model [88], for which the RBF kernel is a good choice [6,7]. The regulation coefficient (Cr) and the kernel parameter (Kp) should be optimised when KELM is applied. KELM was applied using MATLAB and Statistics Toolbox Release 2016a (The MathWorks, Inc., Natick, MA, USA), and the source code was downloaded from http://www.ntu.edu.sg/home/egbhuang/.

### 4.3. Statistical Criteria

The root-mean-square error (RMSE, Equation (4)) and the ratio of performance to deviation (RPD, Equation (5)) [89] were applied to evaluate each method’s estimation accuracy using R version 3.5.3 [74]. Each method was classified into three categories according to RPD values: Category A (RPD > 2.0), Category B (1.4 ≤ RPD ≤ 2.0) and Category C (RPD < 1.4). The models categorised as A or B were assumed to have the potential to estimate chlorophyll content [90]:

(4) $$\mathrm{RMSE}=\sqrt{\frac{1}{n}\overset{n}{\underset{i=0}{\sum }}{\left(\hat{y_{i}}-y_{i}\right)}^{2}}$$

(5) RPD=SD/RMSE

where SD is the standard deviation of the real chlorophyll content, which was calculated from the measurements from the HPLC in the test data, *n* is the number of samples, $y_{i}$ is the real chlorophyll content, and $\hat{y_{i}}$ is the estimated chlorophyll content.

Although RF generates important measures for variables, other algorithms are generally more difficult to implement. Since few studies have attempted cross-algorithm comparisons, a sensitivity analysis of selected narrow-bands of the machine learning algorithm-based regression models using data-based sensitivity analysis (DSA) was conducted. This analysis performs a black-box use of the fitted models with their machine learning algorithms by querying the fitted models with sensitivity samples and recording their responses [91].

## 5. Conclusions

Some stresses are utilised to improve the quality of agricultural products, and the control of light transmission by shade treatment has been conducted to increase chlorophyll content in tea plants. Although chlorophyll content estimation is one of the most common applications of hyperspectral remote sensing, previous studies were based on measurements under relatively low-stress conditions. Therefore, the chlorophyll content estimations based on four algorithms (random forest (RF), support vector machine (SVM), deep belief nets (DBN) and kernel-based extreme learning machine (KELM)) were evaluated using the original reflectance or the first derivative spectra from shade grown tea leaves in this study. The regression models based on KELM and the original reflectance data yielded the most accurate estimations with a root-mean-square error of 8.94 ± 3.05 μg cm^−2^ and the ratio of performance to deviation values from 1.70 to 8.04, which means the regression models based on KELM were excellent for quantifying chlorophyll content.

The advantage of the first derivative spectra was confirmed for only RF but remains obscure for the other three algorithms. Considering that satellite or airborne remote sensing data are preliminary data sources for large-scale assessments (from which the first derivative index is usually unavailable due to spectral resolution), KELM might also be a useful tool for estimating the chlorophyll content of shade-grown tea leaves from satellite- or airborne-based remote sensing data.

## Figures and Tables

**Table 1:** Chlorophyll content (μg cm^−2^) for each treatment and each date.

Sampling Date	TreatMent	Number of Samples	Minimum	Median	Mean	Maximum	Standard Deviation	Skewness	Kurtosis	
10/05/2019	Open	15	44.45	52.55	53.77	68.47	7.22	0.54	−0.86	
10/05/2019	Shaded	15	64.04	75.39	74.92	84.39	6.20	−0.41	−1.11	a, d
12/07/2019	Open	12	31.19	46.36	47.07	60.12	7.43	−0.18	−0.17	a, b
12/07/2019	Shaded	10	37.81	47.93	46.25	53.08	5.89	−0.36	−1.65	b
26/07/2019	Open	15	86.66	115.59	111.93	146.53	20.18	0.01	−1.55	c
26/07/2019	Shaded	10	63.37	95.15	95.32	137.92	19.68	0.54	−0.05	c, d
All		77	31.19	64.80	72.60	146.53	28.08	0.82	−0.25	

Values with the same letter (i.e., a, b, c or d) are not significantly different (*p* < 0.05).

**Table 2:** Ratio of performance to deviation (RPD) and root-mean-square error (RMSE) for each regression model (statistical results are based on 100 repetitions). RF: random forest, SVM: support vector machine, DBN: deep belief nets, KELM: kernel-based extreme learning machine.

	RPD						
	Minimum	Median	Mean	Maximum	Standard Deviation	Skewness	Kurtosis
Original Reflectance							
RF	1.12	1.80	1.87	2.90	0.42	0.21	−0.81
SVM	0.81	2.59	2.69	5.68	1.18	0.44	−0.40
DBN	1.66	2.83	2.99	5.51	0.86	1.04	0.88
KELM	1.70	3.65	3.59	8.05	1.27	0.62	0.33
First derivative spectra							
RF	1.39	2.77	2.79	5.02	0.51	1.01	3.35
SVM	0.59	2.77	2.65	5.92	1.20	0.21	−0.55
DBN	1.27	2.46	2.57	5.62	0.81	1.02	1.85
KELM	1.81	3.47	3.51	5.76	0.88	0.25	−0.47
	RMSE						
	Minimum	Median	Mean	Maximum	Standard deviation	Skewness	Kurtosis
Original reflectance							
RF	9.63	15.31	16.03	27.03	3.66	0.64	0.03
SVM	4.43	10.59	13.34	37.98	7.40	1.47	1.52
DBN	5.28	10.36	10.24	16.77	2.55	0.11	−0.66
KELM	3.68	7.82	8.95	18.25	3.06	0.72	−0.14
First derivative spectra							
RF	6.20	10.39	10.56	18.82	1.91	0.96	3.20
SVM	4.92	10.72	13.91	49.61	8.12	1.51	2.39
DBN	5.26	11.55	12.06	20.72	3.30	0.47	−0.34
KELM	5.22	8.37	8.60	14.32	1.98	0.75	0.23
